# Value of T1 mapping on gadoxetic acid-enhanced MRI for microvascular invasion of hepatocellular carcinoma: a retrospective study

**DOI:** 10.1186/s12880-020-00433-y

**Published:** 2020-04-28

**Authors:** Chenyi Rao, Xinquan Wang, Minda Li, Guofeng Zhou, Hongmei Gu

**Affiliations:** 1 Medical College, Nantong University, Nantong, Jiangsu China; 2 Department of Radiology, Affiliated Hospital of Nantong University, 20 Xisi Rd., Nantong, 226001 Jiangsu China; 3 Department of Radiology, Zhongshan Hospital, Fudan University, Shanghai, China

**Keywords:** Gd-EOB-DTPA, Hepatocellular carcinoma, Microvascular invasion, Magnetic resonance imaging

## Abstract

**Background:**

To evaluate the utility of non-invasive parameters derived from T1 mapping and diffusion-weighted imaging (DWI) on gadoxetic acid-enhanced MRI for predicting microvascular invasion (MVI) of hepatocellular carcinoma (HCC).

**Methods:**

A total of 94 patients with single HCC undergoing partial hepatectomy was analyzed in this retrospective study. Preoperative T1 mapping and DWI on gadoxetic acid-enhanced MRI was performed. The parameters including precontrast, postcontrast and reduction rate of T1 relaxation time and apparent diffusion coefficient (ADC) values were measured for differentiating MVI-positive HCCs (*n* = 38) from MVI-negative HCCs (*n* = 56). The receiver operating characteristic curve (ROC) was analyzed to compare the diagnostic performance of the calculated parameters.

**Results:**

MVI-positive HCCs demonstrated a significantly lower reduction rate of T1 relaxation time than that of MVI-negative HCCs (39.4% vs 49.9, *P* < 0.001). The areas under receiver operating characteristic curve (AUC) were 0.587, 0.728, 0.824, 0,690 and 0.862 for the precontrast, postcontrast, reduction rate of T1 relaxation time, ADC and the combination of reduction rate and ADC, respectively. The cut-off value of the reduction rate and ADC calculated through maximal Youden index in ROC analyses was 44.9% and 1553.5 s/mm^2^. To achieve a better diagnostic performance, the criteria of combining the reduction rate lower than 44.9% and the ADC value lower than 1553.5 s/mm^2^ was proposed with a high specificity of 91.8% and accuracy of 80.9%.

**Conclusions:**

The proposed criteria of combining the reduction rate of T1 relaxation time lower than 44.9% and the ADC value lower than 1553.5 s/mm^2^ on gadoxetic acid-enhanced MRI holds promise for evaluating MVI status of HCC.

## Background

Hepatocellular carcinoma (HCC) ranks the sixth most frequent cancer and the fourth leading cause of cancer-related death worldwide in 2018 [1]. Liver resection, liver transplantation and radiofrequency ablation are the curative treatment modalities for HCC [2]. However, early recurrence is a major problem that impairs the prognosis of HCC patients. Microvascular invasion (MVI) is known as a critical predictor of early recurrence and poor prognosis after curative treatments of HCC [3–5]. A noninvasive evaluation of MVI preoperatively is important because it could potentially affect the surgical choice [4, 5].

In clinical practice, the histology of the surgical specimens after liver resection or transplantation is the only method that can determine the status of MVI. There are some studies that demonstrate the promising results for preoperatively predicting MVI of HCC based on the evaluation of morphologic MR imaging features [6, 7] and the quantitative analysis including measuring the ADC values of diffusion weighted imaging (DWI) [8, 9], kurtosis value of diffusion kurtosis imaging [10] or D value of intravoxel incoherent motion (IVIM) [11]. In DWI model, the ADC value can be measured for reflecting the quantified Gaussian water diffusivity in tissue and better tumor characterization [12]. Recently, a radiomics approach based on radiological images [13] provided satisfactory diagnostic performance for preoperative evaluation of MVI. However, the subjective nature in the evaluation of morphologic features, instable image quality of diffusion weighted/kurtosis images (i.e., T2 blackout effect, susceptibility artifacts and image distortion) or obscure algorithms in radiomics analysis are all challenges for clinical utility.

Gadoxetic acid (Gd-EOB-DTPA) is a liver-specific contrast agent that has been widely accepted for MR imaging with better detection and stage of hepatic nodules [2], and evaluation of liver function in patients with HCC [14]. The signal intensity (SI) on hepatobiliary phase (HBP) during gadoxetic acid-enhanced MRI was determined by the hepatocyte uptake of Gd-EOB-DTPA and hypointense HCC was reported to be associated with more tumor invasiveness and poor outcome [15, 16]. T1 relaxation time measured in a lesion during Gd-EOB-DTPA administration is an absolute value that is more reliable than the SI measurement in reflecting the uptake of Gd-EOB-DTPA [17], because the SI measurement can be affected by some technical factors [18]. Also, T1 mapping can be integrated seamlessly into gadoxetic acid-enhanced MRI to provide high temporal and spatial resolution images with the precontrast, postcontrast T1 relaxation time and its reduction rate (Δ %) calculated. Some previous studies have demonstrated that the parameters of T1 mapping were helpful for predicting histologic grades and recurrence after resection of HCC [19–21].

To our knowledge, few study has reported the role of T1 mapping in assessing MVI of HCC. In addition, DWI combined with T1 mapping is a functional protocol that can be integrated into gadoxetic acid-enhanced MRI. It supposed that the measurement of T1 relaxation time and ADC value is expected to accurately predict MVI of HCC.

The aim of our study was to evaluate whether the parameters derived from T1 mapping and DWI during gadoxetic acid-enhanced MRI can provide a reliable diagnostic performance in evaluation of MVI of HCC.

## Methods

This study was approved by the Institutional Review Board of Zhongshan Hospital, Fudan University (approval number B2018–236) in accordance with the ethical guidelines of the Declaration of Helsinki. The committee waived the requirement for informed consent because it is a retrospective study. All data of patients in our study can be reviewed and analyzed with permissions obtained from the department of radiology and institutional Review Board of Zhongshan Hospital, Fudan University.

### Patients selection

**Fig. 1:**
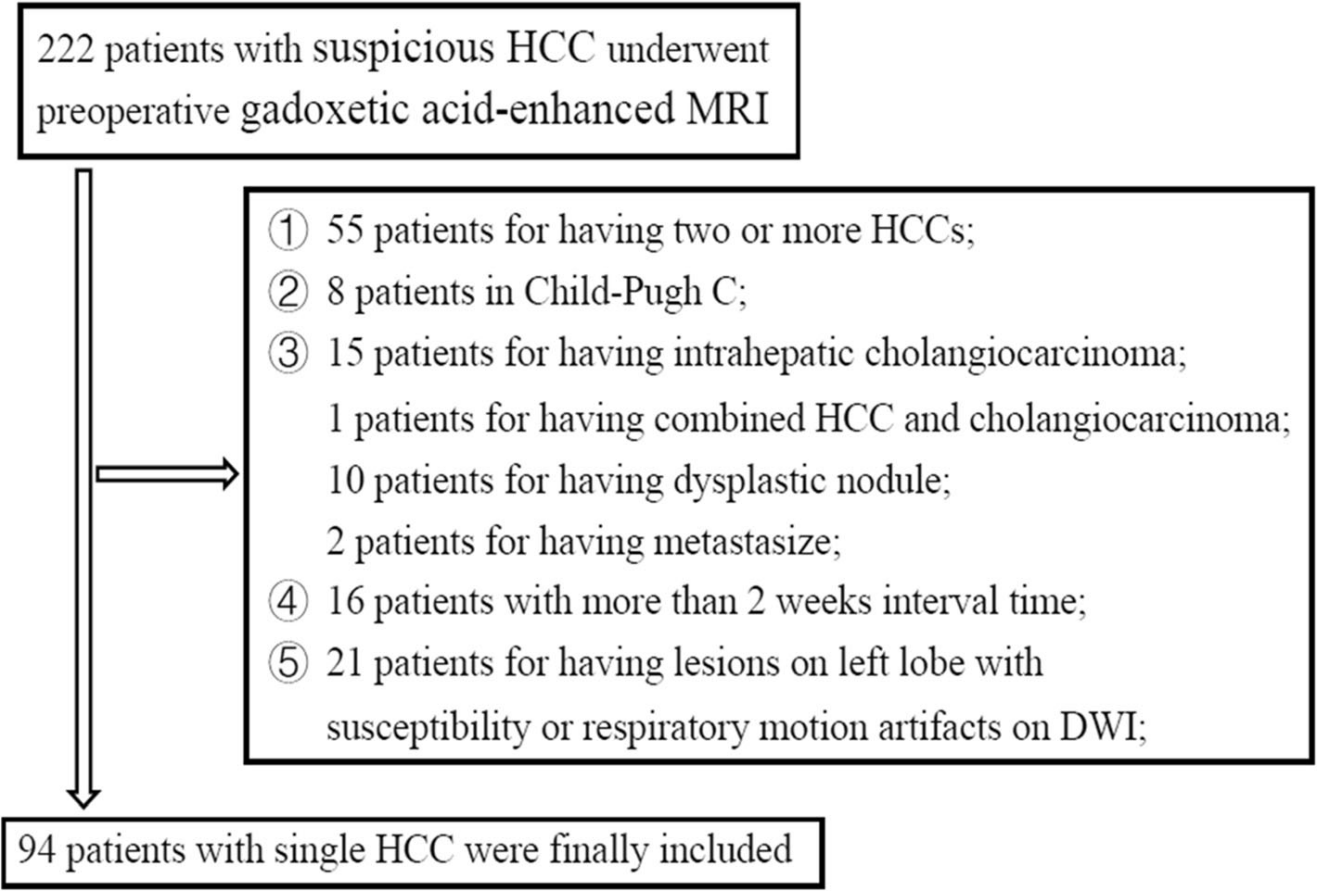
Flow chart of patients selection

According to the 2018 practice guidance by the AASLD [22], patients with preexisting cirrhosis were at high risk for developing HCC and the surveillance program of ultrasound (US) and/or alpha-fetoprotein (AFP) every 6 months were recommended. In our institution, between February 2016 and March 2017, 222 patients underwent gadoxetic acid-enhanced MRI for further evaluation of suspicious HCCs detected during surveillance. Patients with probable benign nodules (i.e., cysts, hemangiomas, arterioportal shunts) screened by US or HCC patients that had macrovascular invasion or receiving any previous treatments such as transcatheter arterial chemoembolization (TACE) and radiofrequency ablation (RFA) were initially excluded from the study. The inclusive criteria (Fig. 1) of the patient selection were: (a) single HCC diagnosed by histology with preoperative gadoxetic acid-enhanced MRI; (b) the interval time between the MRI and the operation in less than 2 weeks; (c) patients in Child-Pugh A-B; (d) having qualified MR images.

### MR imaging protocol

All patients enrolled in our study underwent gadoxetic acid-enhanced MRI in a single 1.5-T MR system (MAGNETOM Aera, Siemens Healthcare, Erlangen, Germany), with a 8-channel phased-array receiver coil. Single-spin echo plane DWI for free breathing (3200/56 milliseconds repetition time (TR)/echo time (TE), 84 × 128 matrix, 380–400 × 300–324 mm field of view (FOV), 5.5 mm slice thickness) was performed, and corresponding ADC maps were automatically generated with b values of 0 and 500 s/mm^2^. Dynamic contrast-enhanced T1-weighted 3D gradient-recalled echo images (3.47 / 1.36 TR / TE, 320 × 195 matrix, 10° flip angle, 308 × 380 mm FOV, 3 mm slice thickness) were obtained after intravenously injection of contrast agent. A dual flip-angle (Flip angle, 2° and 12°) before and at 20 min after injection of gadoxetic acid based on a voxel-by-voxel basis was applied for automatically generating T1 maps with syngo MapIt (Syngo Offline and mono-exponential fit, Siemens Medical Solutions, Erlangen, Germany). The precontrast phase was obtained before a hand bolus injection of 0.025 mmol/kg of gadoxetic acid (Primovist, Bayer Schering Pharma, Berlin, Germany) at a rate of 1 ml/sec with a subsequent 20 ml saline flush. Subsequent MR images during the arterial phase (automatically triggered when the ascending aorta reached peak enhancement), the portal vein phase (about 14 s), the transition phase (about 3 min), and the hepatobiliary phase (HBP; 20 min) were obtained.

### MR images analysis

**Fig. 2:**
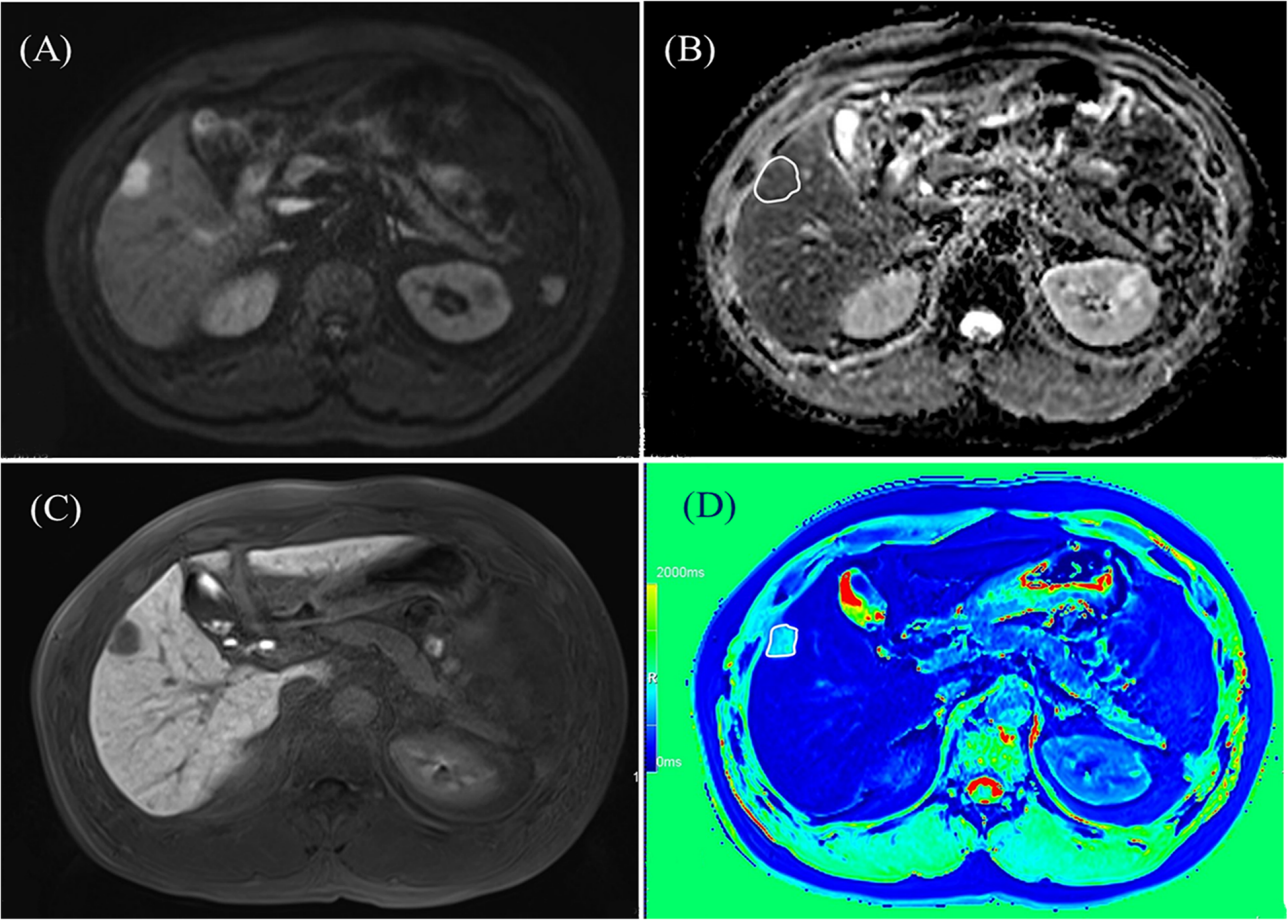
The ROIs of HCC was drawn on the corresponding ADC maps and postcontrast T1 maps. **a** the HCC on right lobe of liver showing hyperintensity on DWI maps of b = 500 mm^2^/s; **b** ROI was drawn on corresponding ADC maps; **c** the HCC on right lobe of liver showing hypointensity on hepatobiliary phase; **d** ROI was drawn on postcontrast T1 maps

Two abdominal radiologists (C.Y.R. with 5 years of experience and X.Q.W. with 10 years of experience) independently reviewed the MR images. Both the two radiologists knew that all the patients were having HCC but they were blinded to the histology of MVI. The region of interest (ROI) in a lesion was outlined around the edge of tumor on each slice on the precontrast T1 maps, hepatobiliary phase T1 maps and the ADC maps by the two radiologists. The ROIs were copied from the same ROIs that drawn on the high flip angle (12°) T1-weighted images and high b value (500 s/mm^2^) images, respectively (Fig. 2). Precontrast and postcontrast T1 relaxation time were obtained from the precontrast T1 maps and hepatobiliary phase T1 maps, respectively. The ADC values of HCCs were also calculated by the two observers. The reduction rate (Δ%) of T1 relaxation time were calculated by using the following formula: Δ% = 100% × (pre T⁠1 value − post T⁠1 value)/pre T⁠1 value [14, 20], in which the pre T⁠1 and post T⁠1 values representing the T⁠1 relaxation time before and after the injection of gadoxetic acid.

### Reference standard for MVI

Pathological data including presence of cirrhosis, Edmondson-Steiner grade of I, II, III and IV according to the nuclear grading scheme [23] or microvascular invasion was according to surgical pathologic reports generated by our institutional pathologists specialized in liver histology (each individual with more than 20 years of experience). Microvascular invasion was defined as presence of tumor invasive in any portal vein, hepatic vein, or a large capsular vessels lined by endothelium that was visible only microscopically.

### Statistical analysis

Frequencies of categorical variables for differentiating MVI were compared by using Fisher exact test. Difference of quantitative variables including precontrast/postcontrast T1 relaxation time and ADC values between MVI-positive and MVI-negative groups was compared by using independent sample t test. The interclass correlation coefficient (ICC) of quantitative data between the two observers was calculated (poor: < 0.40; fair: 0.40–059; good: 0.60–0.74; excellent: 0.75–1.00). The multivariate logistic regression analysis was used to achieve a predictive model of combining the reduction rate and the ADC. Area under receiver operating characteristic curve (AUC) with 95% confidence interval (95% CI) based on receiver operating characteristic curve (ROC) analysis for each parameter and the combined model was generated for evaluating the utility of variables to discriminate the status of MVI. Sensitivity, specificity, accuracy, positive predictive value (PPV), negative predictive value (NPV) and likelihood ratio (LR) of appropriate cut-off value corresponding to maximal Youden index by using ROC analysis and of some optional threshold values were calculated with 95% CI. All the statistical tests were performed by using statistical software (SPSS version 21, SPSS, Chicago, III) and a two-side *P* value less than 0.05 indicating significance level.

## Results

### Patients and treatment characteristics

Among the 222 patients, 55 patients were excluded for having two or more HCCs; 8 patients were excluded for Child-Pugh C because the poor liver function could impair the quality of gadoxetic acid-enhanced MR images; 28 were excluded for other types of nodules including intrahepatic cholangiocarcinoma (*n* = 15), combined HCC and cholangiocarcinoma (*n* = 1), dysplastic nodule (*n* = 10) and metastases (*n* = 2); 16 patients were excluded for having more than 2 weeks interval time during follow-up; 21 patients were excluded for having lesions on left lobe with susceptibility or respiratory motion artifacts on DW images. All the patients underwent partial hepatectomy for HCC. Finally, a total of 94 patients (76 men and 18 women; median age: 54 years (range, 24–75 years) with single HCC were included in the study. Among the 94 HCC patients, 87 patients (92.6%) were hepatitis B virus infected. The mean size and standard deviation of the maximal diameter of tumors (ranging from 1.2 to 4.5 cm) measured on HBP was 1.9 cm and 0.8 cm. According to the histology, 56 HCCs showed presence of MVI and 38 HCCs showed absence of MVI. The detailed characteristics of patients are shown in Table 1.

**Table 1:** Baseline characteristics of patients with hepatocellular carcinoma

Characteristics	All patients (*n* = 94)	MVI		*P value*
		Negative (*n* = 56)	Positive (*n* = 38)	
Gender				0.217
Male	88 (93.6%)	54 (57.4%)	34 (36.2%)	
Female	6 (6.4%)	2 (2.1%)	4 (4.3%)	
Age, years (range)	52 ± 11 (24–75)	52 ± 10 (30–73)	53 ± 12 (24–75)	0.744
Diameter, cm (range)	1.9 ± 0.8 (1.2–4.5)	1.8 ± 0.8 (1.2–3.5)	2.2 ± 0.7 (1.4–4.5)	0.078
Etiology				0.190
HBV	87 (92.6%)	54 (57.4%)	33 (35.1%)	
HCV	5 (5.3%)	1 (1.1%)	4 (4.2%)	
Alcohol or none	2 (2.1%)	1 (1.1%)	1 (1.1%)	
Presence of cirrhosis				0.754
Presence	66 (70.2%)	40 (42.6%)	26 (27.7%)	
Absence	28 (29.8%)	16 (17.0%)	12 (12.7%)	
Edmondson-Steiner grade				0.380
I-II	57 (60.6%)	36 (38.3%)	21 (22.3%)	
III-IV	37 (39.4%)	20 (21.3%)	17 (18.1%)	
AFP > 20 ng/ml				> 0.99
Positive	51 (54.3%)	30 (31.9%)	21 (22.3%)	
Negative	43 (45.7%)	26 (27.7%)	17 (18.1%)	
Child-Pugh				> 0.99
A	91 (96.8%)	54 (57.4%)	37 (39.4%)	
B	3 (3.2%)	2 (2.1%)	1 (1.1%)	

*HBV* Hepatitis B virus, *HCV* Hepatitis C virus, *AFP* a-fetoprotein, *MVI* Microvascular invasion

### T1 relaxation time and ADC measurements

**Fig. 3:**
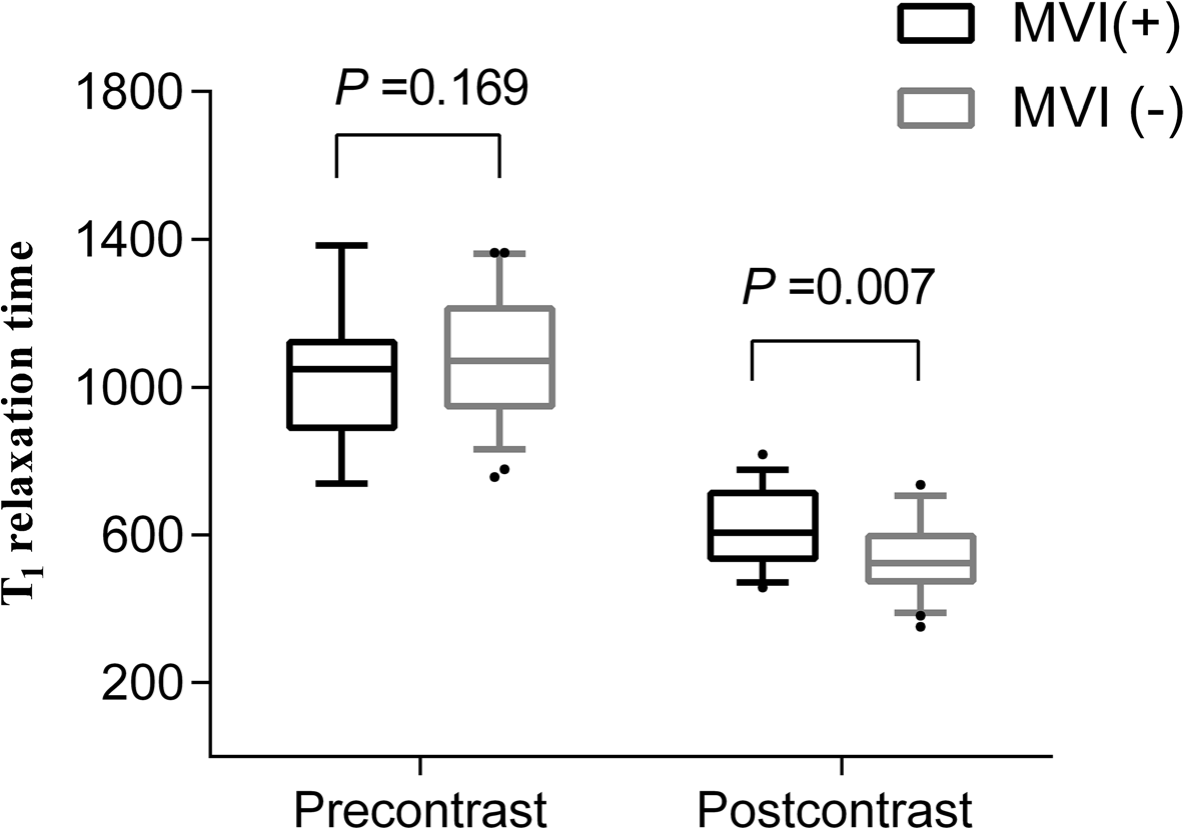
Comparisons of mean values and standard deviation of precontrast and postcontrast T1 relaxation time between MVI-positive and MVI-negative HCCs. Each box shows the mean values and 25th and 75th percentiles. • represents outliers of more than 95th percentiles

**Fig. 4:**
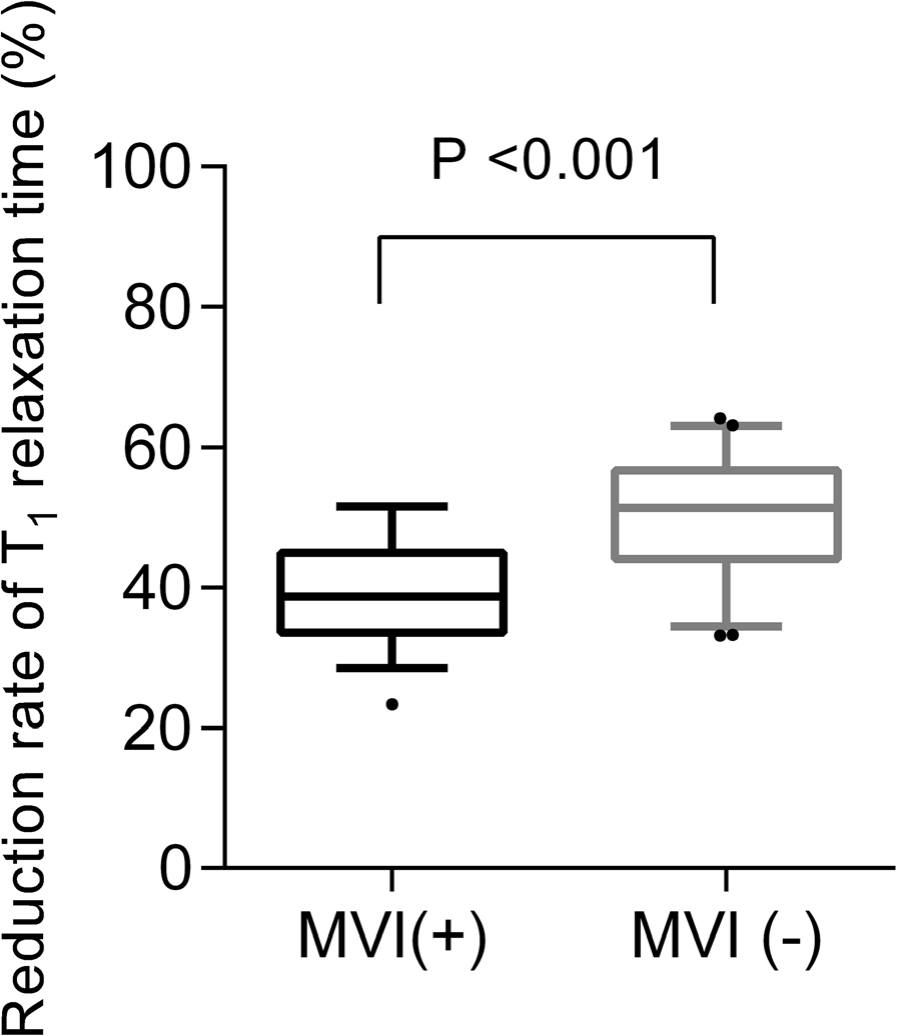
Comparisons of mean values and standard deviation of reduction rate T1 relaxation time between MVI-positive and MVI-negative HCCs. The box shows the mean value and 25th and 75th percentiles. • represents outliers of more than 95th percentiles

Table 2 shows the mean values of T1 relaxation time (precontrast, postcontrast) and ADC values measured by the two observers. The reduction rates were then calculated based on the mean precontrast and postcontrast T1 relaxation time of the two observers. As shown in Fig. 3, there was no statistically significant difference for precontrast T1 relaxation time between MVI-negative and MVI-positive groups. The mean value of postcontrast T1 relaxation time of the two observers were significantly higher in MVI-positive HCCs than that in MVI-negative HCCs (621.0 ms vs 536.5 ms, *P* < 0.001). The reduction rates of T1 relaxation time was significantly lower in MVI-positive HCCs than that in MVI-negative HCCs (39.4% vs 49.9, *P* < 0.001, Fig. 4). The mean ADC value was significantly lower in MVI-positive HCCs than that in MVI-negative HCCs (1.495 × 10^−^ 3 mm^2^/s vs 1.620 × 10^−^ 3 mm^2^/s, *P* = 0.003). The agreements between the two observers shown in Table 2 were excellent for ADC, precontrast and postcontrast T1 relaxation time (ICC: 0.759, 95% CI: 0.637–0.840; ICC: 0.823, 95% CI: 0.744–0.879; ICC: 0.858, 95% CI: 0.786–0.906, respectively).

**Table 2:** Comparisons of mean values and standard deviations of T1 relaxation time and apparent diffusion coefficient (ADC) value between MVI-negative and MVI-positive groups of hepatocellular carcinoma

	All (*n* = 94)	MVI		*P* value	ICC (95% CI)^a^
		Negative (*n* = 56)	Positive (*n* = 38)		
T1 relaxation time					
Pre-contrast					0.823 (0.744–0.879)
Observer 1	1040.9	1065.9	1004.2	0.095	
SD	175.6	191.2	144.3		
Observe 2	1079.2	1094.2	1057.1	0.283	
SD	163.5	149.9	181.4		
Mean	1060.1	1080.0	1030.6	0.148	
SD	161.9	163.6	156.9		
Postcontrast					0.858 (0.786–0.906)
Observe 1	578.1	548.5	621.7	0.001	
SD	107.9	102.3	102.2		
Observe 2	563.2	524.5	620.3	< 0.001	
SD	117.1	103.8	113.3		
Mean	570.6	536.5	621.0	< 0.001	
SD	105.4	95.6	99.8		
Reduction rate (%) ^a^	45.7	49.9	39.4	< 0.001	
SD	9.2	8.0	7.1		
ADC					0.759 (0.637–0.840)
Observe 1	1571.5	1597.4	1533.3	0.134	
SD	194.4	175.6	216.0		
Observe 2	1568.7	1643.9	1457.8	< 0.001	
SD	257.1	233.6	252.6		
Mean	1570.1	1620.7	1495.6	0.003	
SD	204.6	194.3	198.8		

*MVI* Microvascular invasion, *SD* Standard deviations, *CI* Confidence intervals, *ICC* Interclass correlation coefficient, *ADC* Apparent diffusion coefficient

^a^ The ICC was not calculated because reduction rate of T1 relaxation was based on the mean values of precontrast and postcontrast T1 relaxation time of the two readers

### Diagnostic performance for evaluating MVI of HCC

**Fig. 5:**
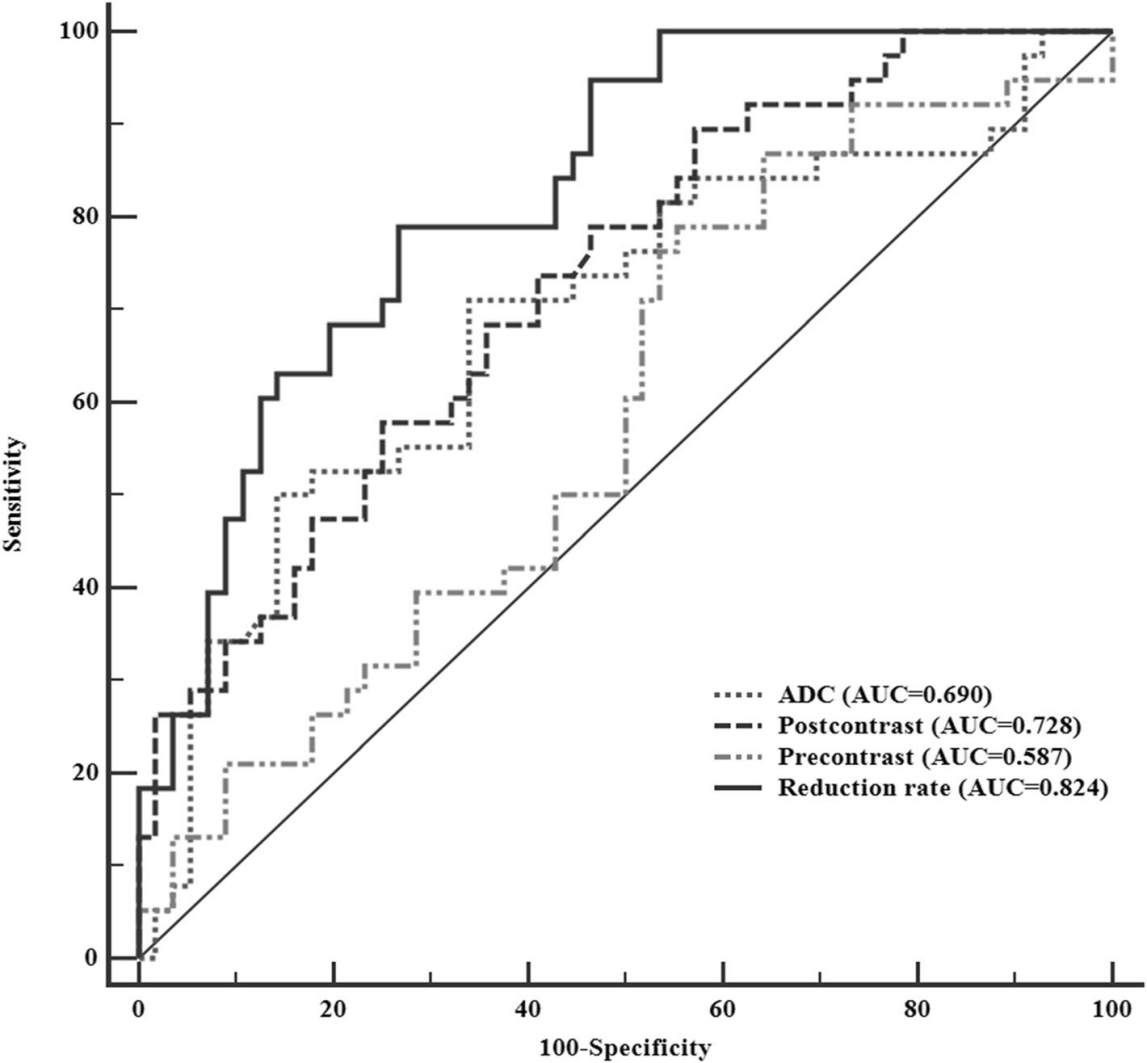
The utility of receiver operating characteristic curve of precontrst, postcontrast and reduction rate of T1 relaxation time, the ADC value and the combination of the reduction rate and the ADC value to discriminate MVI-positive and MVI-negative HCCs

The AUCs in ROC analyses were compared for the diagnostic performance among the parameters driving from T1 relaxation time and DWI in evaluation of MVI status of HCC (Fig. 5). The corresponding AUC, cut-off value, sensitivity, specificity, +LR, −LR, PPV and NPV with 95% CI were summarized in Table 3. We also used the multivariate logistic regression analysis to achieve a combined predictive model by entering the two significant parameters of the reduction rate and the ADC. The precontrast, postcontrast, reduction rate of T1 relaxation time, the ADC and the combination model of reduction rate and ADC demonstrated an AUC of 0.587, 0.728, 0.824, 0.690 and 0.862, respectively, for predicting MVI of HCC. Among each parameter of T1 relaxation time and the ADC value, the reduction rate was the most reliable feature that showed the highest AUC of 0.824 in ROC analyses. However, after combining the reduction rate and the ADC, the AUC of the combined model (0.862) was achieved even higher. The cut-off value (44.9%) of the reduction rate in the ROC analyses for predicting MVI showed a sensitivity, specificity and accuracy of 79.0, 73.2 and 75.5%, respectively. The AUC of reduction rate of T1 relaxation time was significantly higher than that of ADC (*P* = 0.043).

**Table 3:** Diagnostic performance for T1 relaxation time and ADC value in evaluating MVI-positive HCC

Parameters	AUC	Thresholds	Sensitivity	Specificity	Accuracy	+LR	-LR	PPV	NPV
T1 relaxation time									
Pre-contrast	0.587	1138.4^a^	78.9%	44.7%	58.5%	1.4	0.5	49.2%	75.8%
95% CI	0.481–0.688		62.7–90.4%	313–58.5%	48.6–68.5%	1.1–1.9	0.2–0.9	36.1–62.3%	57.7–88.9%
		862.1^b^	21.1%	91.1%	62.8%	2.4	0.9	61.5%	63.0%
			9.6–37.3%	80.4–97.0%	52.3–72.5%	0.8–6.7	0.7–1.0	31.6–86.1%	51.5–73.4%
Postcontrast	0.728	586.7^a^	57.9%	75.0%	68.1%	2.3	0.6	61.1%	72.4%
95% CI	0.626–0.814		40.8–73.7%	61.6–85.6%	58.7–77.5%	1.4–3.9	0.40–0.80	43.5–76.9%	59.1–83.3%
		679.4^b^	34.2%	91.1%	68.1%	3.8	0.7	72.2%	67.1%
			19.6–51.4%	80.4–97.0%	58.7–77.5%	1.5–9.9	0.6–0.9	46.5–90.3%	55.4–77.5%
Reduction rate	0.824	44.9%^a^	79.0%	73.2%	75.5%	3.0	0.3	66.7%	83.7%
95% CI	0.732–0.895		62.7–90.4%	59.7–84.2%	66.9–84.2%	1.9–4.7	0.2–0.5	51.0–80.0%	70.3–92.7%
		38.0%^b^	47.4%	91.1%	74.4%	5.3	0.6	78.3%	71.8%
			31.0–64.2%	80.4–97.0%	64.5–82.3%	2.2–13.1	0.4–0.8	56.3–92.5%	59.9–81.9%
ADC	0.690	1553.5^a^	71.1%	66.1%	68.1%	2.1	0.4	58.7%	77.1%
95% CI	0.586–0.781		51.1–84.6%	52.2–78.2%	58.7–77.5%	1.4–3.2	0.30–0.70	43.2–73.0%	62.7–88.0%
		1339.1^b^	31.6%	92.9%	69.1%	4.8	0.7	76.5%	67.5%
			17.5–48.7%	82.7–98.0%	59.8–78.5%	1.7–13.6	0.6–0.9	50.1–93.2%	55.9–77.8%
Reduction rate + ADC	0.864^d^	44.9% + 1553.5^c^	60.6%	91.8%	80.9%	7.4	0.4	80.0%	81.2%
95% CI	0.776–0.952		42.2–76.6%	81.2–96.9%	72.9–88.9%	3.1–17.9	0.3–0.7	58.7–92.4%	69.6–89.2%

*AUC* Area under receiver operating characteristic curve, *LR* Likelihood ratio, *PPV* Positive predictive value, *NPV* Negative predictive value, *CI* Confidence intervals, *ADC* Apparent diffusion coefficient

^a^ represents the cut-off values calculated though maximal Youden index in ROC analysis

^b^ represents the optional thresholds that demonstrating a specificity just higher than 90% with a reliable higher sensitivity

^c^ represents a proposed criteria of combining the reduction rate lower than 44.9% and the ADC value lower than 1553.5 s/mm^2^ that demonstrated the highest diagnostic accuracy of predicting MVI of HCC

^d^ represents the AUC in ROC analysis was calculated based on the model of combining the reduction rate and the ADC in multivariate logistic regression model

As shown in Table 3, in the ROC analyses, we also calculated the optional threshold values of the parameters of T1 mapping and DWI that demonstrated a high specificity just over 90%with a relative higher sensitivity. An optional threshold value (38%) of the reduction rates showed the highest sensitivity of 47.4% and the same high specificity of 91.1% among that of parameters of T1 mapping. An optional threshold value (1339.1 s/mm^2^) of ADC values showed a sensitivity of 31.6% and a high specificity of 92.9%. To achieve better diagnostic performance, we proposed a criteria of combining the reduction rate lower than 44.9% and the ADC value lower than 1553.5 s/mm^2^ that can demonstrate a satisfactory diagnostic accuracy of 89.9% with a sensitivity of 60.6% and a high specificity of 91.8%.

## Discussion

The study demonstrated that MVI-positive HCC could show significantly higher postcontrast T1 relaxation time and reduction rate than MVI-negative HCC. The reduction rate of T1 relaxation time demonstrated a better diagnostic performance for predicting MVI status of HCC in comparison with that of ADC. To achieve a high diagnostic accuracy, we also proposed a criteria of combining the reduction rate and ADC with the cut-off values calculated.

Gadoxetic acid-enhanced MRI combined with DWI is a part of the standard workup in detection and characterization of hepatic nodules for better providing clinicians with roadmap of therapeutic strategies in our institution. We quantitatively compared the parameters derived from T1 mapping of HCC to the ADC value with good image quality for identifying the MVI status of HCC. The previous studies reported that the ADC value derived from DWI was useful in evaluation of MVI status of HCC [8, 9, 24]. In line with our study, a lower ADC value was helpful for predicting MVI of HCC because theoretically it reflects higher tissue cellularity and decreased micro-capillary perfusion [9]. There are several pitfalls of DWI that could affect the reliability of the ADC measurement including [25, 26]: *(1)* limited image quality with poor signal-to-noise ratio and low spatial resolution; *(2)* more sensitive to motion and air susceptibility, especially for pulsation artifacts in left robe; *(3)* misregistration artifacts on ADC map; *(4)* T2 blackout effect mainly due to fibrotic tissues or calcifications depicting hypointesity on both DW images and ADC maps. Some studies reported that the reproducibility for ADC and IVIM measurement of hepatic nodules was poor [27–29].

T1 mapping can be used as an additional protocol integrated in the sequences of gadoxetic acid-enhanced MR imaging for evaluation of diffuse liver disease [14, 30, 31]. Ding [30] et al. reported that the measurement of T1 relaxation time was more reproducible compared with the measurement of ADC values for staging hepatic fibrosis. Previous studies also showed that T1 mapping outperformed DWI during gadoxetic acid-enhanced MRI for evaluation of liver function in patients with HCC [14] and staging hepatic fibrosis [30, 31].

The reduction rate of T1 relaxation time demonstrated improved diagnostic performance compared with that of ADC values even after excluding the patients that showed moderate to evident artifacts of tumor on DW images. In our institution, the sequence of T1 mapping with Syngo MapIt is routinely performed, which can provide the acquisition of MR images with a high resolution 3D-dataset of whole liver. Our results demonstrated that there was no statistical significance in predicting MVI of HCC by using precontrast T1 relaxation time because it may be affected by some factors such as liver inflammation [32]. Our results indicated that a lower reduction rate of T1 relaxation time was a potential predictor for MVI positive status of HCC. Peng [19] et al. reported that the reduction rate in T1 value was the best predictor for the differentiation of HCC and higher histological grade of HCC is correlated with MVI positive status of HCC [33]. Wang [20] et al. demonstrated that reduction rate of T1 relaxation time was a reliable biomarker for predicting recurrence of HCC (≤ 3 cm) after hepatectomy. The SI on HBP during gadoxetic acid-enhanced MRI has a shorten T1 effect that determined by expression levels of the organic anion transporter 1B3 (OATP8) protein in HCC, and was reported to have a strong association with the expression of Wnt/β-catenin target genes [34]. Additionally, in contrast to the HCC with overexpression of OATP8, HCC with β-catenin gene mutations showed more aggressiveness in tumor biology and an increased probability of MVI [35, 36]. Hence, MVI-positive HCC may show higher SI on HBP and lower reduction rate of T1 relaxation time.

In our study, the parameters of T1 mapping and DWI demonstrated the limited diagnostic performance for predicting MVI of HCC because all the specificities of the cut-off values corresponding to maximal Youden index were low, which could lead to more false-positive incidences. We also showed an optimal threshold value in ROC analysis with a high specificity of over 90%. However, as the specificity increased, the sensitivity was decreased to be acceptable. An ADC value of DWI can help improve the diagnostic accuracy of MVI of HCC [2], however, the reliability of measurement can also be affected by some technical factors. We further proposed a criteria of combining the reduction rate of T1 relaxation and ADC value by using their cut-off value corresponding to maximal Youden index, which demonstrated a significant increased sensitivity of 60.6% and a comparable high specificity of 91.8%.

The present study is limited by its selection bias of retrospective nature. The sample size of the study is relative small. Additionally, we only used two b-values of 0, 500 s/mm^2^ for DW imaging that are routinely performed in our institution. The measurement of ADC values based on these two b values was previously applied for evaluating MVI of small HCCs with satisfactory diagnostic performance [24]. In our study, to reduce the measurement error, we excluded patients having DW images with moderate to evident artifacts, especially those showing artifacts caused by pulsation artifacts in the left lobe, misregistration and air susceptibility.

## Conclusions

Currently, MVI of HCC can only be confirmed by histology and a noninvasive approach to MVI status of HCC for guiding tumor management, such as selecting appropriate allocation of liver transplantation [4] and resection margin [5] is limited. So far, there is a large body of evidence that gadoxetic acid-enhanced MRI integrating DWI and T1 mapping can show a high accuracy for characterization of focal liver lesions and evaluation of the whole and segmental liver function reserve. Our results suggested that the criteria of combining T1 relaxation time and ADC on gadoxetic acid-enhanced MRI holds promise to provide additional information for MVI status of HCC, which is preliminary and warrants further validation.

## Data Availability

The datasets used and/or analysed during the current study are available from the corresponding author on reasonable request.

## Abbreviations

MVI: Microvascular invasion
HCC: Hepatocellular carcinoma
DWI: Diffusion-weighted imaging
ROC: Receiver operating characteristic curve
ADC: Apparent diffusion coefficient
Gd-EOB-DTPA MRI: Gadoxetic acid-enhanced magnetic resonance imaging
HBP: Hepatobiliary phase
US: Ultrasound
AFP: a-fetoprotein
TACE: Transcatheter arterial chemoembolization
RFA: Radiofrequency ablation
TR: Repetition time
TE: Echo time
FOV: Field of view
ROI: Region of interest
ICC: Interclass correlation coefficient
AUC: Area under receiver operating characteristic curve
PPV: Positive predictive value
NPV: Negative predictive value
LR: Likelihood ratio
OATP8: Organic anion transporter
